# Acquired Angioedema Associated With Systemic Lupus Erythematosus Presenting as Acute Abdomen: A Case Report

**DOI:** 10.1002/jgf2.70116

**Published:** 2026-04-24

**Authors:** Seiji Shiota, Shogo Kodama, Kouichirou Tahara

**Affiliations:** ^1^ Department of General Medicine, Faculty of Medicine Oita University Yufu Oita Japan; ^2^ Department of Endocrinology, Metabolism, Rheumatology and Nephrology, Faculty of Medicine Oita University Yufu Oita Japan; ^3^ Department of Surgery Kunisaki City Hospital Kunisaki Japan

**Keywords:** acquired angioedema, acute abdomen, systemic lupus erythematosus

## Abstract

Acquired angioedema (AAE) due to C1 inhibitor deficiency can present as acute abdomen. A 24‐year‐old woman developed severe abdominal pain and bowel wall edema initially suggestive of hereditary angioedema, but genetic testing excluded it. She later manifested fever, arthritis, rash, and serological abnormalities consistent with systemic lupus erythematosus (SLE). SLE‐associated AAE was diagnosed, and immunosuppressive therapy led to clinical and biochemical improvement. This case highlights the importance of considering AAE in patients with unexplained abdominal pain and hypocomplementemia to avoid misdiagnosis and unnecessary surgical intervention.

## 1 Background

Acquired angioedema (AAE) due to C1 inhibitor (C1‐INH) deficiency is a rare disorder characterized by recurrent non‐urticarial edema involving the skin, airway, and gastrointestinal tract [1]. Unlike hereditary angioedema (HAE), AAE occurs secondary to autoimmune or lymphoproliferative diseases and is mediated by excessive bradykinin production [1]. When the bowel is affected, patients often present with severe abdominal pain, bowel wall thickening, and ascites, findings that closely mimic acute abdomen and may result in unnecessary laparotomy [2]. Here, we describe a case of AAE associated with systemic lupus erythematosus (SLE) presenting as acute abdomen. In patients with unexplained abdominal pain, bowel wall edema, and hypocomplementemia, clinicians should consider AAE associated with SLE and initiate appropriate immunosuppressive therapy, thus avoiding unnecessary surgery.

## 2 Case Presentation

**FIGURE 1:**
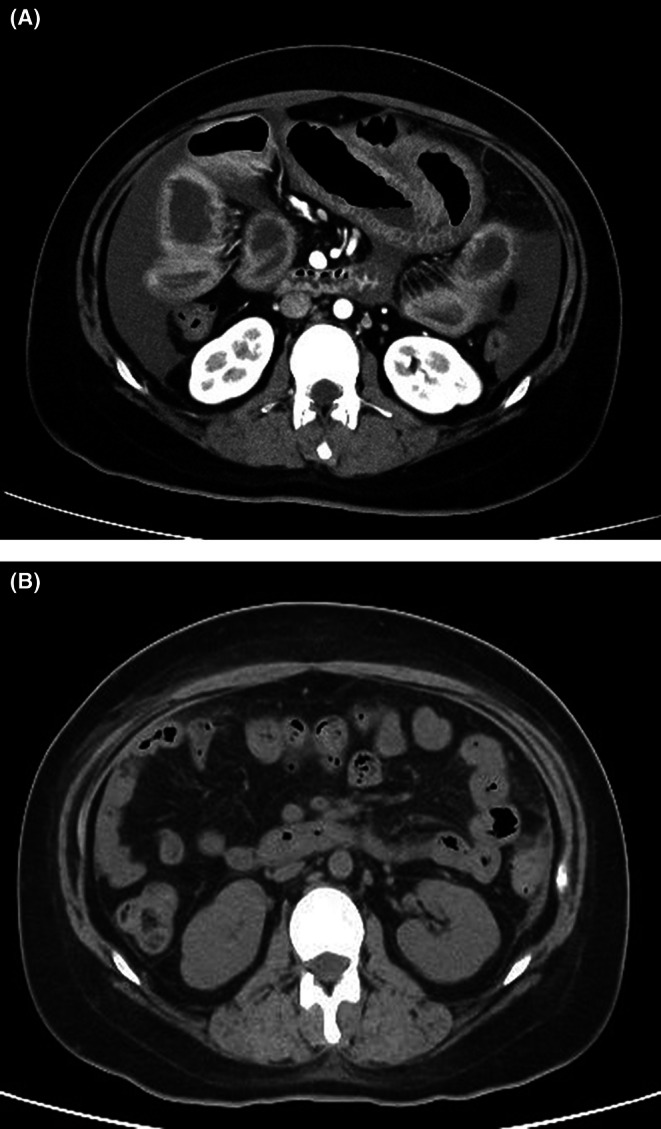
(A) Abdominal contrast‐enhanced computed tomography showed diffuse edema of the small bowel wall with ascites, raising the suspicion of angioedema or lupus enteritis. (B) Follow‐up CT images demonstrated the resolution of intestinal edema after treatment.

**FIGURE 2:**
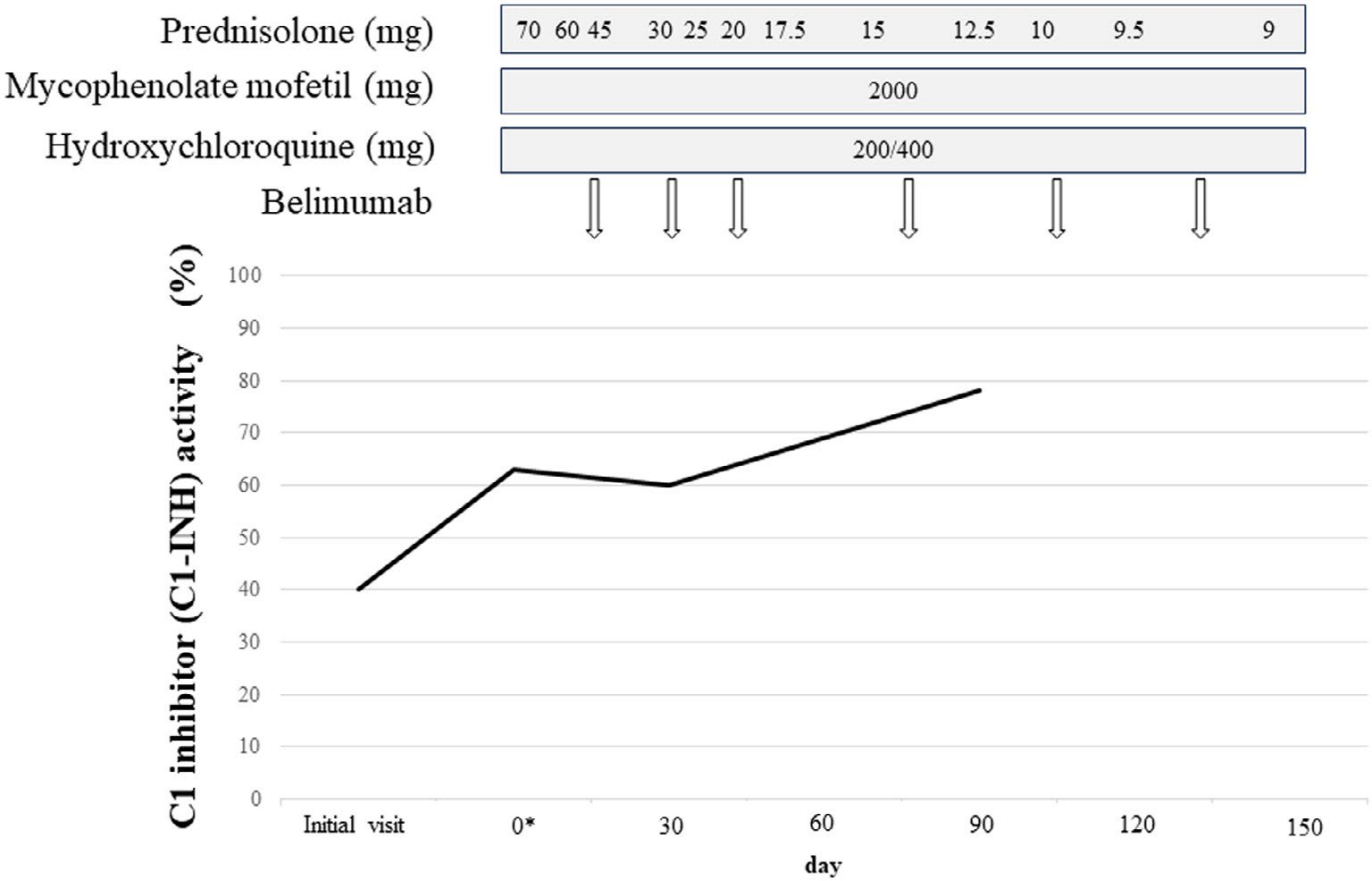
Time course of immunosuppressive therapy for systemic lupus erythematosus (SLE) and serial changes in C1 inhibitor (C1‐INH) activity. Day 0 represents the start of treatment. C1‐INH activity progressively increased in parallel with clinical improvement. Numbers represent the daily dose of prednisolone (PSL, mg/day), indicating dose tapering during treatment. Hydroxychloroquine was administered at 200 and 400 mg on alternate days. Belimumab was administered at 10 mg/kg at weeks 0, 2, and 4, followed by every 4 weeks.

A 24‐year‐old woman presented with progressive abdominal pain, nausea, and vomiting. She had a history of herpes zoster meningitis at age 17 but no allergies, no family history of angioedema, and no smoking or alcohol use. On admission, her height was 153 cm and weight 77 kg. Her temperature was 37.2°C, blood pressure 110/83 mmHg, heart rate 95 beats/min, and oxygen saturation 95% on room air. Physical examination revealed abdominal distension with normal bowel sounds and tenderness in the epigastric and lower quadrants without rebound tenderness or guarding. No edema or rash was noted on the extremities or face. Abdominal contrast‐enhanced computed tomography (CT) showed diffuse edema of the small bowel wall with ascites. There were no imaging findings suggestive of perforation, intestinal ischemia, or intestinal obstruction, and therefore, emergency surgical intervention was not indicated, and conservative management was appropriately selected (Figure 1A). Laboratory tests revealed hypocomplementemia and reduced C1‐INH activity (40%) (Table 1), raising the suspicion of HAE. During hospitalization, she developed lip swelling, intermittent fever, arthralgia, and erythematous plaques on both elbows and knees. Her abdominal pain gradually improved during the same period. Further tests revealed leukopenia, antinuclear antibody positivity, elevated anti‐dsDNA and anti‐Sm antibodies, and persistently low complement levels. Based on the clinical manifestations (fever, arthritis, skin rash, and leukopenia) and immunological findings (positive antinuclear antibody, anti–double‐stranded DNA antibody, anti‐Sm antibody, and hypocomplementemia), the patient fulfilled the 2019 ACR/EULAR classification criteria for systemic lupus erythematosus with a total score of 35 points and was therefore diagnosed with SLE. Because genetic analysis revealed no pathogenic variants in the SERPING1 gene and the serum C1q level was markedly decreased (5.3 mg/dL), HAE was excluded, and the patient was diagnosed with AAE associated with SLE. The patient had a SLEDAI score of 38, indicating severe disease activity. According to current guidelines [3], steroid pulse therapy (methylprednisolone 125 mg/day for 2 days) was initiated, followed by treatment with oral prednisolone (70 mg/day), mycophenolate mofetil, and hydroxychloroquine. In addition, belimumab, a B‐cell modulator that selectively inhibits B lymphocyte stimulator and reduces autoreactive B cells involved in the pathogenesis of SLE, was administered at regular intervals. After initiation of immunosuppressive therapy, clinical manifestations of SLE gradually improved. Along with clinical improvement, C1‐INH activity progressively normalized. The longitudinal changes in C1‐INH activity are shown in Figure 2. Follow‐up CT images demonstrated the resolution of intestinal edema after treatment (Figure 1B). She was discharged in a stable condition for 3 months after her hospitalization. After discharge, the dose of prednisolone was gradually tapered, and belimumab has been administered every 4 weeks as maintenance therapy.

**TABLE 1:** Laboratory findings.

Item	Results	Reference range	Unit
CRP	0.7	0–0.14	mg/dL
Total protein	9.1	6.6–8.1	g/dL
Albumin	3.3	4.1–5.1	g/dL
Total bilirubin	1.4	0.4–1.5	mg/dL
AST	34	13–30	U/L
ALT	26	10–42	U/L
ALP	52	38–113	U/L
γ‐GTP	23	9–32	IU/L
BUN	13.3	8–20	mg/dL
Creatinine	0.4	0.6–1.0	mg/dL
Sodium	130	138–145	mmol/L
Potassium	3.9	3.6–4.8	mmol/L
Chloride	93	101–108	mmol/L
Blood glucose	122	73–109	mg/dL
Red blood cell count	5.56	4.35–5.55	×million/μL
Hemoglobin	13.0	13.7–16.8	g/dL
Mean corpuscular volume	73.4 L	83.6–98.2	fL
White blood cell count	3.6	3.3–8.6	×1000/μL
Neutrophil	37.2	38.5–80.5	%
Lymphocyte	55.7	16.5–49.5	%
Eosinophil	0.6	0.0–8.5	%
Platelet count	244	158–348	×1000/μL
C3	25.7	73–138	mg/dL
C4	3.4	11–31	mg/dL
CH50	0.4	31.6–57.6	U/mL
C1 inhibitor activity	40	70–130	%

Abbreviations: γ‐GTP, γ‐glutamyltransferase; ALP, alkaline phosphatase; ALT, alanine aminotransferase; AST, aspartate aminotransferase; BUN, blood urea nitrogen; CRP, C‐reactive protein.

## 3 Discussion

AAE due to C1‐INH deficiency is a rare and potentially life‐threatening disorder. Angioedema is a self‐limiting, nonpitting edema of the skin and mucous membranes [2, 4]. It most commonly presents as painless, nonpruritic swelling of the lips, face, and extremities, but may also involve the larynx or gastrointestinal tract [2, 4]. According to a systematic review, the most common sites of involvement in AAE were the face (67.0%), followed by the upper airway (63.3%), abdomen (55.0%), extremities (45.9%), and genitalia (11.0%) [1]. Our patient's presentation initially suggested HAE; however, the absence of a significant family history and negative genetic testing ruled out hereditary disease. Instead, the coexistence of systemic manifestations and serological markers confirmed SLE‐associated AAE. This condition is exceedingly rare; while HAE occurs in about 2% of SLE patients, fewer than 1% develop AAE [5], and only a limited number of such cases have been reported worldwide [6]. Plasma‐derived C1‐INH are effective and widely used as a first‐line therapy for acute HAE attacks [7]. In contrast, AAE results from the secondary consumption or inactivation of C1‐INH. The absence of SERPING1 mutations together with a decreased serum C1q level is helpful in distinguishing AAE from HAE [8]. Previous studies have shown that AAE is frequently associated with underlying diseases, including lymphoproliferative disorders, monoclonal gammopathy, autoimmune diseases, and solid tumors [1]. Because of this association, identifying SLE is essential for determining the appropriate treatment strategy in patients with AAE. Failure to investigate SLE may result in delayed diagnosis and inappropriate management, including unnecessary surgical intervention or insufficient treatment of the underlying autoimmune disease. To achieve sustained normalization of C1‐INH activity and prevent recurrence of angioedema, the underlying disease must be accurately identified and appropriately treated [1]. Therefore, distinguishing AAE from HAE and conducting a comprehensive evaluation for secondary causes are critical for optimal management.

Differentiating lupus enteritis from intestinal angioedema, although important from a treatment perspective, is particularly challenging. Both conditions may present with bowel wall edema and ascites and demonstrate similar CT findings, such as “target” or “comb” signs [9]. Lupus enteritis arises from small‐vessel vasculitis and typically responds to corticosteroids, whereas intestinal angioedema is bradykinin‐driven and is related to complement consumption. In this case, persistently reduced C1‐INH activity supported the diagnosis of AAE.

Awareness of this entity is critical, as gastrointestinal angioedema may be the initial manifestation of SLE and can mimic surgical emergencies [10]. Prompt recognition of this condition helps prevent unnecessary surgery and guides appropriate therapy. Our patient responded well to immunosuppressive treatment, with normalization of C1‐INH activity and resolution of angioedema, consistent with previous reports [6].

## 4 Conclusion

In case of the patients with unexplained abdominal pain with bowel wall edema, clinicians should consider AAE. Examination of complements should be considered in young women with unexplained abdominal symptoms and autoimmune features. Early recognition and targeted immunosuppression are essential to avoid misdiagnosis and improve patient outcomes.

## Author Contributions

All authors contributed to study conception and design. S.S. was responsible for collecting and analyzing the data and drafted the manuscript. S.K., K.T. contributed to collecting the data and participated in the conceptualization of the manuscript. All authors have read and approved the final manuscript.

## Funding

The authors have nothing to report.

## Ethics Statement

Written informed consent was obtained from the patient for publication of this case report. Institutional review board approval was not required for this case report according to the authors' institutions.

## Conflicts of Interest

The authors declare no conflicts of interest.

## Data Availability

The data that support the findings of this study are available from the corresponding author upon reasonable request.
